# Interferon Regulatory Factor-1 (IRF1) activates autophagy to promote liver ischemia/reperfusion injury by inhibiting β-catenin in mice

**DOI:** 10.1371/journal.pone.0239119

**Published:** 2020-11-02

**Authors:** Bing Yan, Jing Luo, Christof Kaltenmeier, Qiang Du, Donna B. Stolz, Patricia Loughran, Yihe Yan, Xiao Cui, David A. Geller

**Affiliations:** 1 Department of Surgery, University of Pittsburgh, Pittsburgh, PA, United States of America; 2 Department of Hepatobiliary and Pancreatic Surgery, The First Affiliated Hospital of Zhengzhou University, Zhengzhou, Henan, PR China; 3 Center for Biologic Imaging, University of Pittsburgh Medical School, Pittsburgh, PA, United States of America; National Institutes of Health, UNITED STATES

## Abstract

Autophagy is an important factor in liver ischemia-reperfusion injury. In the current study we investigate the function of interferon regulatory factor-1 (IRF1) in regulating autophagy to promote hepatic ischemia reperfusion injury (IR). The high expression of IRF1 during hepatic IR exhibited increased liver damage and was associated with activation of autophagy shown by Western blot markers, as well as immunofluorescent staining for autophagosomes. These effects were diminished by IRF1 deficiency in IRF1 knock out (KO) mice. Moreover, the autophagy inhibitor 3-MA decreased IR-induced liver necrosis and markedly abrogated the rise in liver injury tests (AST/ALT). β-catenin expression decreased during liver IR and was increased in the IRF1 KO mice. Immunoprecipitation assay showed the binding between IRF1 and β-catenin. Overexpression of IRF1 induced autophagy and also inhibited the expression of β-catenin. β-catenin inhibitor increased autophagy while β-catenin agonist suppressed autophagy in primary mouse hepatocytes. These results indicate that IRF1 induced autophagy aggravates hepatic IR injury in part by inhibiting β-catenin and suggests that targeting IRF1 may be an effective strategy in reducing hepatic IR injury.

## Introduction

Liver ischemia-reperfusion injury (IR) is involved in hypoxic liver organ stress followed by reperfusion injury after re-oxygenation [1, 2]. Hepatic IR causes liver dysfunction postoperatively and is seen after liver resection or liver transplantation [3–5]. Studying the early signaling events accounting for liver damage associated with IR may provide strategies to mitigate liver injury.

Autophagy is an intracellular process where impaired proteins or organelles are phagocytosed and transported to lysosomes for degradation [6]. Low level autophagy helps cells to satisfy energy demands in periods that include hypoxia [7]. In contrast, excessive autophagy can be triggered by an acute insult such as IR and result in degradation of essential organelles and may lead to cell death [8–11]. Regulating autophagy pathways may ameliorate the damaging effects of IR.

IRF1 is a master transcription factor triggered by type I and II IFNs to activate gene expression of many IFN-responsive genes. IRF-1 plays a harmful role in liver IR [12, 13], as well as reperfusion injury in other organs [12, 14, 15]. More recently, IRF-1 KO mice studies have shown that inhibiting autophagy was protective in hepatic IR [16, 17].

β-catenin has a vital signaling function in many cancer types including hepatocellular carcinoma and colon cancer [26]. However, the role of β-catenin in regulating IRF1 mediated liver IR and effect on autophagy has not been evaluated. The study aims to understand whether IRF-1 is a modulator of autophagy during hepatic IR. Moreover, the effects of IRF1 expression on β-catenin activity, autophagy, and liver injury were examined. Our data indicates that IRF-1 is an activator of autophagy thereby aggravating hepatic IR by inhibiting β-catenin.

## Material and methods

### Ethics

Mouse warm IR and hepatocytes isolation were performed using protocols approved by University of Pittsburgh Institutional Animal Care and Use Committee (IACUC).

### Animal studies

Male C57BL/6J wild type (WT) and IRF-1 knock out (KO) (B6.129S2-Irf1tm1Mak/J, IRF-1-/-) mice were obtained from Jackson Laboratory, 8–12 weeks old and weighed 22-28g. Animals were maintained under strict guidelines of the University of Pittsburgh. The protocol was approved by the University of Pittsburgh Institutional Animal Care and Use Committee (Protocol Number: 17019893). The mice were housed in a constant temperature, laminar-flow, specific pathogen-free environment and had access to food and water ad libitum.

### Isolation of murine hepatocytes and culture

Hepatic parenchymal cells were isolated from murine liver using collagenase digestion, as previously described [15]. The liver was perfused through vena cava with Perfusate I solution (0.14mM NaCl, 6.7mM KCl, 10mM HEPES, 0.22uM EGTA, pH 7.5) at a flow rate of 10ml/min. Next, the liver was perfused with 0.5% collagenase (Worthington) in Perfusate I. After perfusion, the hepatic cells were suspended in William E Media (Thermo) and filtrated through a 700-μm nylon mesh. The hepatic parenchymal cells were separated by centrifugation at 4°C at 400g for 5min.

### Warm liver I/R

A non-lethal model of segmental (70%) hepatic warm I/R was used as previously described [18]. The mouse was anesthesia with ketamine/xylazine (100 mg/kg and 10 mg/kg i.p.). The portal to the left and median liver lobes were clamped with microvascular clamp for 60 min, then removed the microvascular clamp and restored blood flow for 3h,6h,12h,24h. At the end, the animals were anesthetized with inhaled isoflurane, then sacrificed to collect serum and liver samples.

### Liver function tests

Liver function tests following IR were performed using a veterinary chemistry analyzer (model 4000 Drichem, HESKA, Loveland, CO) to measure serum alanine aminotransferase (ALT).

### Isolation of nuclear and cytoplasmic protein

Frozen liver tissues or cells were suspended in cold PBS containing 0.1% Triton X-100 and lysed by homogenization. The mixture was centrifuged at 1000g for 5 min at 4°C and the supernatant stored at -80°C for analysis of cytoplasmic proteins. Nuclear proteins were extracted using a premade buffer containing 20 mM Tris (pH 7.5), 20% glycerol, 420 mM NaCl, 0.2 mM EDTA, 1.5 mM MgCl_2_ and 0.1% Triton X-100. Following a centrifugation step at 13,000g for 15 min at 4°C, nuclear proteins were obtained. Protein concentration was quantitated with BCA assay reagent (Thermo).

### Cell proliferation assay

Hepatocyte function was determined using a commercially available CCK-8(MCE) kit. Cells were plated at a density of 10^4^ cells/well in 96 well plate and cultured overnight. CCK-8 solution was added for 1h at 37°C. The absorbance was measured at 450 nm using a plate reader (Bio-Tek Instruments Inc, Winooski, Vt).

### Adenovirus transfection in vitro and in vivo

Adenovirus was transfected in vitro using the protocol as follows: cells were plated in 10 cm plates and recovered overnight. Culture media was removed and 4ml media that contains adenovirus was added with adenoviral concentration of 50 MOI. Six hours later, another 2 ml of culturing media was added. Two days after transfection, cells were used for testing. Adenoviral IRF-1 (AdIRF-1) is used as previously described [19, 20]. The adenoviral vector encoding lacZ is use as control.

### Drugs

Bafilomycin A1 (Autophagy inhibitor), XAV939 (β-catenin inhibitor) and SKL2001(β-catenin agonist) were from MedChemExpress.

### Real-time PCR

Total RNA from liver and hepatocytes was extracted by Trizol reagent (Invitrogen). 2ug of total RNA was reversely transcribed into cDNA with RNA to cDNA Ecodry Premix Kit (Takara). Then, PCR was performed using SYBR Premix Kit (Takara) on an ABI Stepone PCRSystem (Applied Biosystems). The relative expression of mRNA was quantitated using the 2-ΔΔCt method and normalized to β-actin. Primers used were: β-actin Forward 5`tgttaccaactgggacgaca3`, Reverse5`ggggtgttgaaggtctcaaa3`; β-catenin Forward 5`TGGACCCTATGATGGAGCATGA3`, Reverse5`GGTCAGTATCAAACCAGGCCAG3`

### Western blot

A commercially available lysis buffer was used to extract cellular proteins (Cell Signaling). β-actin or Histone H3 protein was detected as standardization of whole cell protein or nuclear protein respectively. The membrane was scanned by Li-Cor Odyssey. Antibodies used were as follows: antibodies against IRF1, LC3A/B, Beclin1and P62 (Cell signaling technology); β-actin and Histone H3 (Abcam); β-catenin (BD biosciences). The results of Western Blot was quantified by Image Studio (Ver 5.2) software (Li-Cor). β-actin and Histone H3 were used for standardization.

### Co-immunoprecipitation assay

Hepatocytes cells were infected with IRF1-adenovirus. After 48 h, cells were incubated on ice and lysed in cold lysis buffer (Cell Signaling) adding Protease Inhibitor Cocktail (Med Chem Express). Lysates were centrifuged at 12000g at 4°C for 10 min and incubated with anti-IRF1 antibody or anti-β-catenin antibody attached to Protein A/G(Santa Cruz) agarose overnight at 4°C on a shaker platform. Beads were collected by centrifugation at 3000g for 5 min at 4°C, washed in lysis buffer and resuspended in SDS gel loading buffer. The proteins were analyzed by Western blot.

### Immunofluorescence assay

Cells were treated with IRF1-adenovirus and LacZ-adenovirus (as control) and were fixed with 2% paraformaldehyde for 15 min. The cells were incubated with LC3 antibody (Novus) and Cy3-conjugated goat anti-rabbit IgG (Jackson Immunoresearch). F-actin phalloidin (Invitrogen) and Hoechst nuclear stain was also applied. Imaging conditions were maintained at identical settings within each antibody-labeling experiment with original gating performed using the negative control. Large area images in X and Y were obtained using a Nikon A1 confocal microscope.

### Statistical analysis

All experiments were performed in triplicates. Mean standard deviation was calculated for all experiments and shown within the results. Using Student t test or one-way analysis of variance (ANOVA) was used to determine significance. A level of *P* < 0.05 was considered statistically significant.

## Results

### IRF1 expression is increased during warm IR and contributes to liver injury

**Fig 1:**
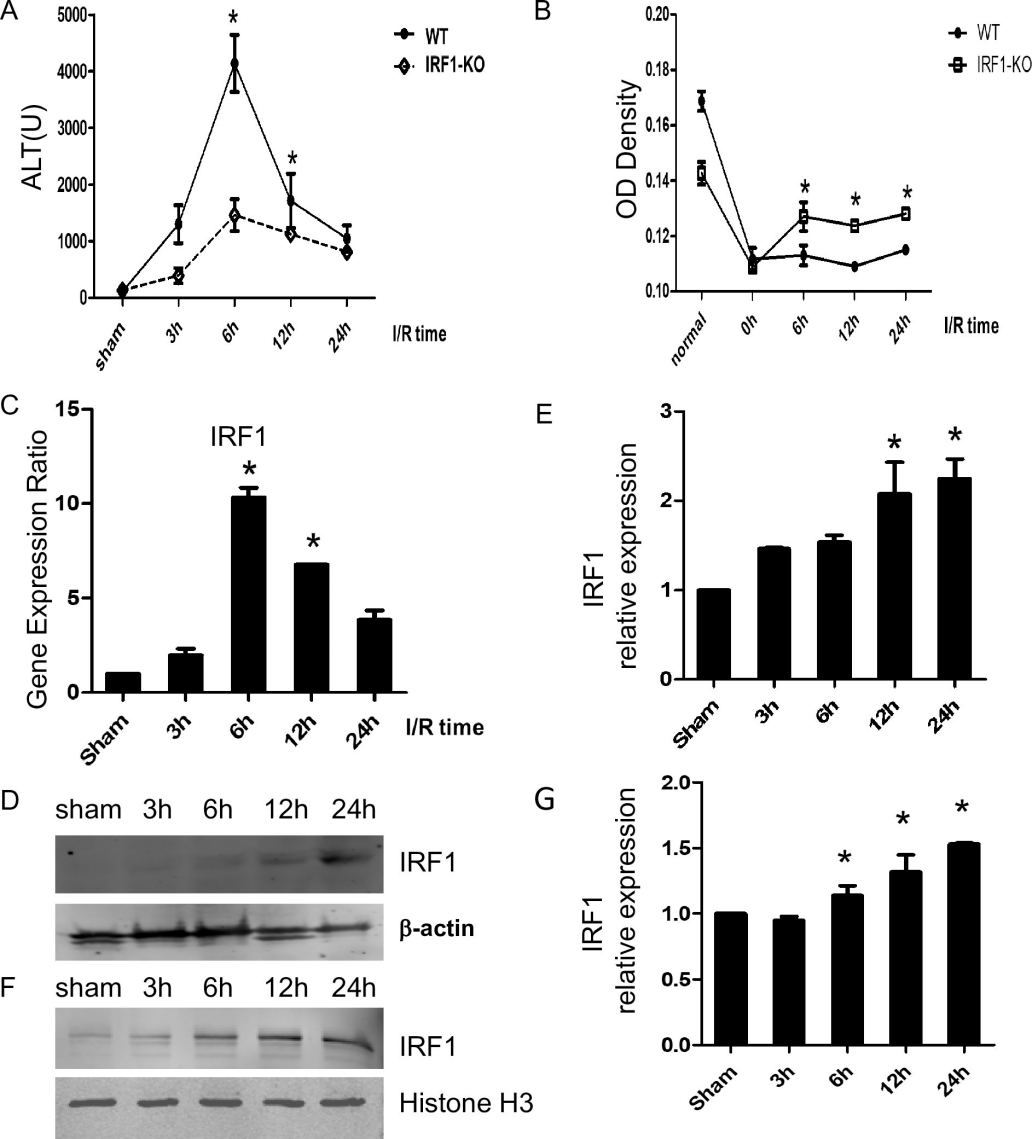
IRF1 up-regulation do harm for hepatocytes in warm IR. A) The serum ALT levels were significantly increased in WT mice after exposure to IR as compared with the IRF1-KO mice(n = 6). B) The cell growth was detected by CCK-8 assay in hypoxia reoxygenation treatment. C) IRF1 gene expression increased during IR. D and E) IRF1 cytoplasmic (D and E) and nuclear (F and G) protein expression was tested by Western Blot and densitometric analysis(n = 3). Data are presented as mean±SD, *P< 0.05 vs. the sham group.

To determine the effect of IRF1 on warm hepatic IR, we used IRF1-KO or wild-type B6 mice to evaluate the changes in liver damage. Serum ALT levels increased significantly (p < 0.05) in wild-type mice and peaked at 6 hr after reperfusion (Fig 1A). Compared with WT, serum ALT decreased in IRF1-KO mice. We also performed RT-PCR and Western blot of whole liver tissue in IR to determine IRF1 expression. Hepatic IRF-1 mRNA was significantly up-regulated during IR in a time-dependent manner (Fig 1C). Likewise, IRF1 cytoplasmic and nuclear protein expression was significantly induced following IR with levels peaking 6–12 hrs after IR (Fig 1D–1G). To test the effects of IRF1 on hepatocyte growth, isolated mouse hepatocytes from WT or IRF1 KO mice were exposed to 1% hypoxia for 1 hour, then re-oxygenated. Hepatocyte growth increased 6–24 hrs after re-oxygenation in the IRF1-KO hepatocytes compared to WT (Fig 1B), suggesting that endogenous IRF1 inhibits cell growth. These data show that IRF1 is harmful in vitro and in vivo during liver IR.

### Autophagy is decreased in IRF1 KO mice during warm IR

**Fig 2:**
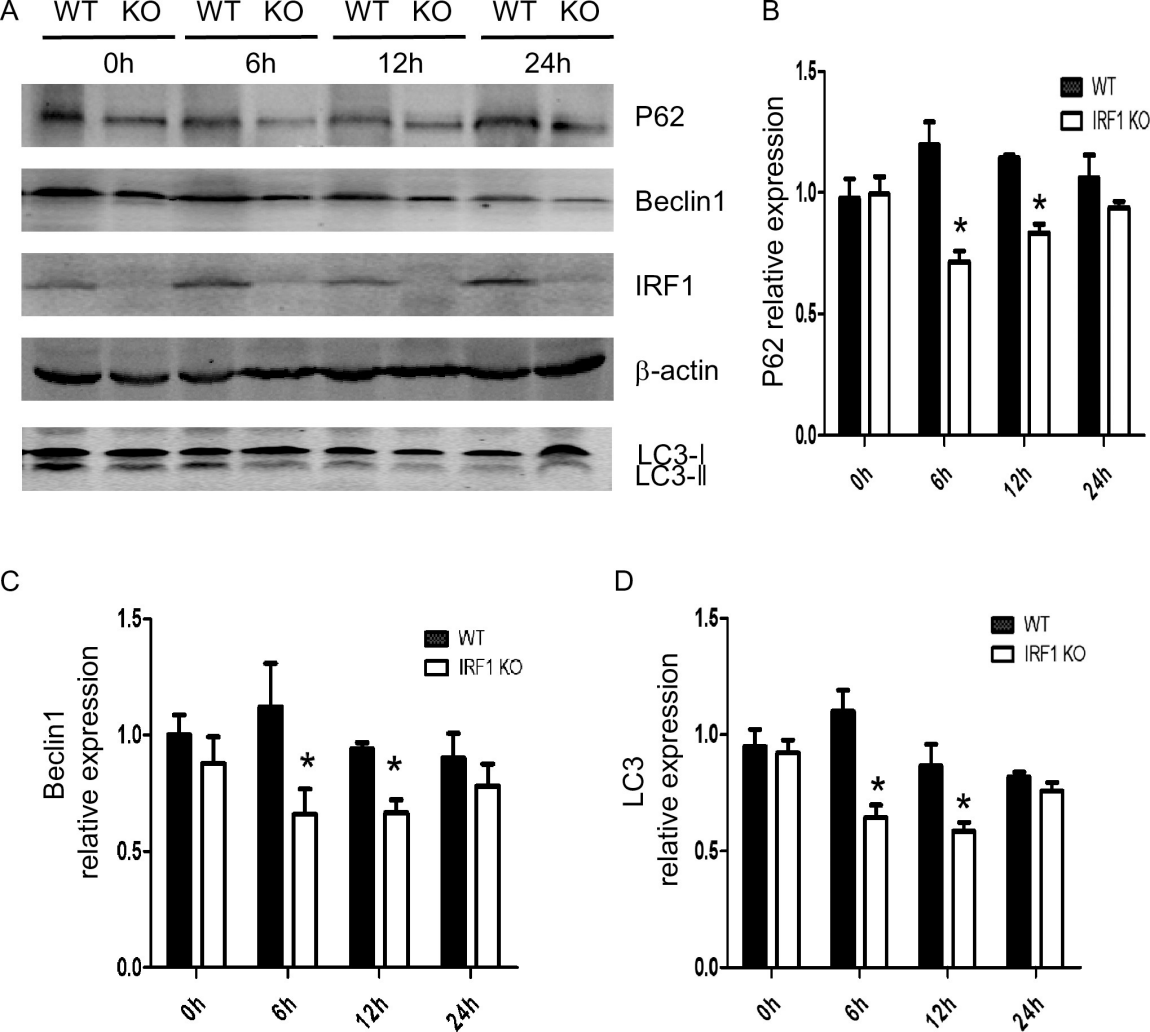
IRF1 induced autophagy and apoptosis in warm IR. A) The proteins of p62, beclin1 and LC3 were detected by Western Blot in livers from WT and IRF1-KO mice (6, 12, 24 h after reperfusion) and densitometric analysis of the relative expression of p62(B), Beclin1 (C) and LC3 (D). n = 3, data are presented as mean±SD, *P< 0.05 vs. the WT group.

To determine the role of IRF1 in autophagy in warm IR, we evaluate the changes in autophagic signaling. Autophagy proteins p62 and beclin1 are decreased at 6 and 12 hrs after warm IR in the IRF1 KO mice, but not in the WT mice (Fig 2A–2C). IRF1 protein is induced 6–24 hrs after IR in WT, but not the IRF KO mice. Likewise, autophagy marker LC3A/B ratio was decreased at 6 and 12 hrs after IR in the IRF1 KO compared to WT mice (Fig 2D). These results indicate that IRF1 deficiency decreases autophagy proteins, and that hepatic autophagy induced by warm IR is mediated in part by IRF-1. To confirm that autophagy was associated with liver injury, we inhibited autophagy with 3-MA (autophagy inhibitor) and found liver necrosis was attenuated, and serum ALT and AST markedly decreased compared to control mouse in IR 6 hrs (S1 Fig).

### β-catenin is increased in IRF KO mice during IR

**Fig 3:**
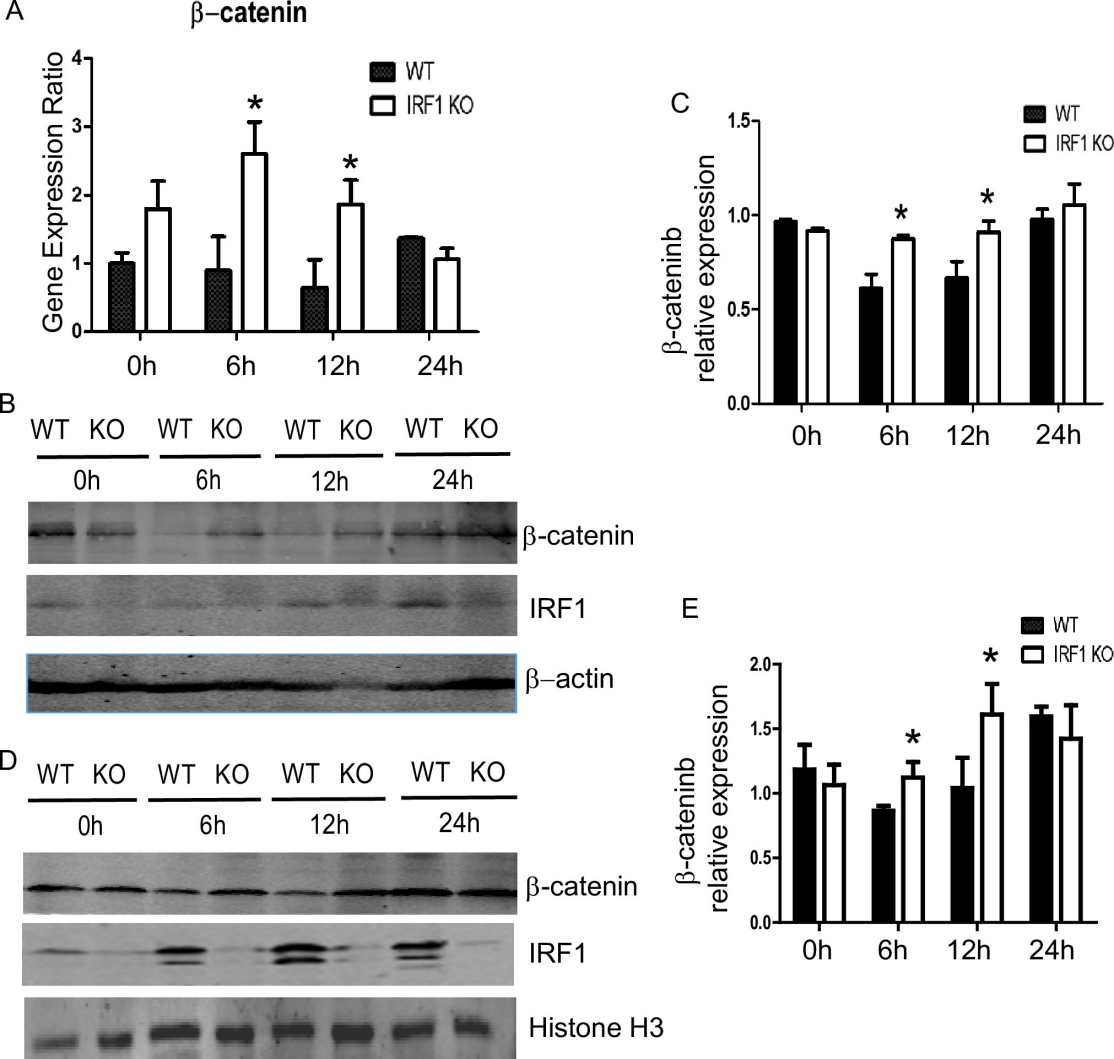
β-catenin increased in IRF1-KO mouse. A) β-catenin mRNA expression in livers from WT and IRF1-KO mice (6, 12, 24 h after reperfusion) was detected by RT-PCR. Western blot for cytoplasmic (B and C) and nuclear (D and E) β-catenin protein. n = 3 per group, data are presented as mean±SD, *P< 0.05 vs. the WT group.

The Wnt/β-catenin pathway is an important regulator of autophagy. To examine the effect of IRF1 on β-catenin during hepatic warm IR, β-catenin gene expression was measured by Western blot 6–24 hrs after warm IR. Compared to WT mice, the IRF-1 KO mice had markedly increased β-catenin mRNA levels at 6 and 12 hrs after warm IR (Fig 3A). Likewise, increased β-catenin protein level was seen 6–12 hrs after reperfusion in the IRF1 KO mice compared to WT mice (Fig 3B–3E). These findings suggest that endogenous IRF1 in the WT mice inhibits β-catenin expression because the β-catenin levels were increased in the IRF1 KO mice.

### IRF1 co-immunoprecipitation with β-catenin

**Fig 4:**
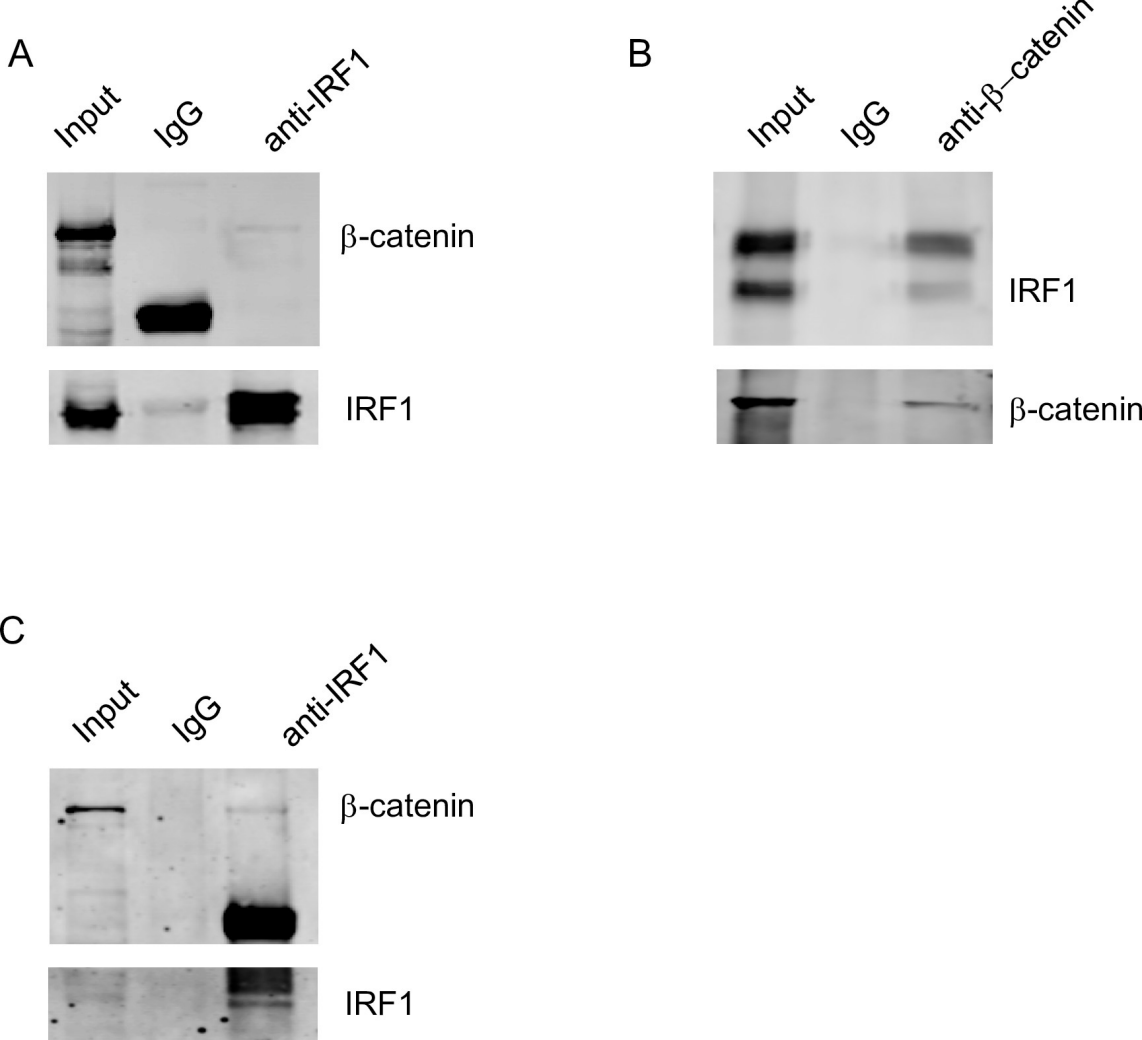
IRF1 has immunoprecipitation with β-catenin. A) Co-IP assay of primary liver cells transfected with adenoviral-IRF1. The interactions of IRF1 with β-catenin were determined by pulling down with anti-IRF1 antibody and Western blotting with anti-β-catenin antibody. B) Co-IP assay of primary liver cells transfected with adenoviral-IRF1. Anti-β-catenin antibody and Western blotting with anti-IRF1 antibody showed antibody to β-catenin pulled down IRF-1. C) Co-IP assay of liver tissue 6 hours after warm IR with anti-IRF1 antibody pulling down β-catenin.

Next, to determine if IRF1 can directly bind β-catenin protein, we used co-immunoprecipitation assay to test the binding between IRF1 and β-catenin proteins. IRF1 was overexpressed in normal mouse hepatocytes using adenoviral-IRF1 transduction, and co-immunoprecipitation pull-down assay showed that antibody to IRF1 bound β-catenin (Fig 4A). Control IgG antibody did not pull down β-catenin. Successful expression of AdIRF1 was confirmed by Western blot for IRF1 (Fig 4A). Next, antibody to β-catenin (but not IgG) pulled down IRF1 protein (Fig 4B). β-catenin expression in the mouse hepatocytes was confirmed by Western blot (Fig 4B). We also performed co-IP pulldown assay looking at liver tissue 6 hrs after hepatic IR injury. We detected the direct binding of β-catenin to IRF1 after murine warm IR (Fig 4C).

### IRF1 inhibits β-catenin expression in primary liver cells

**Fig 5:**
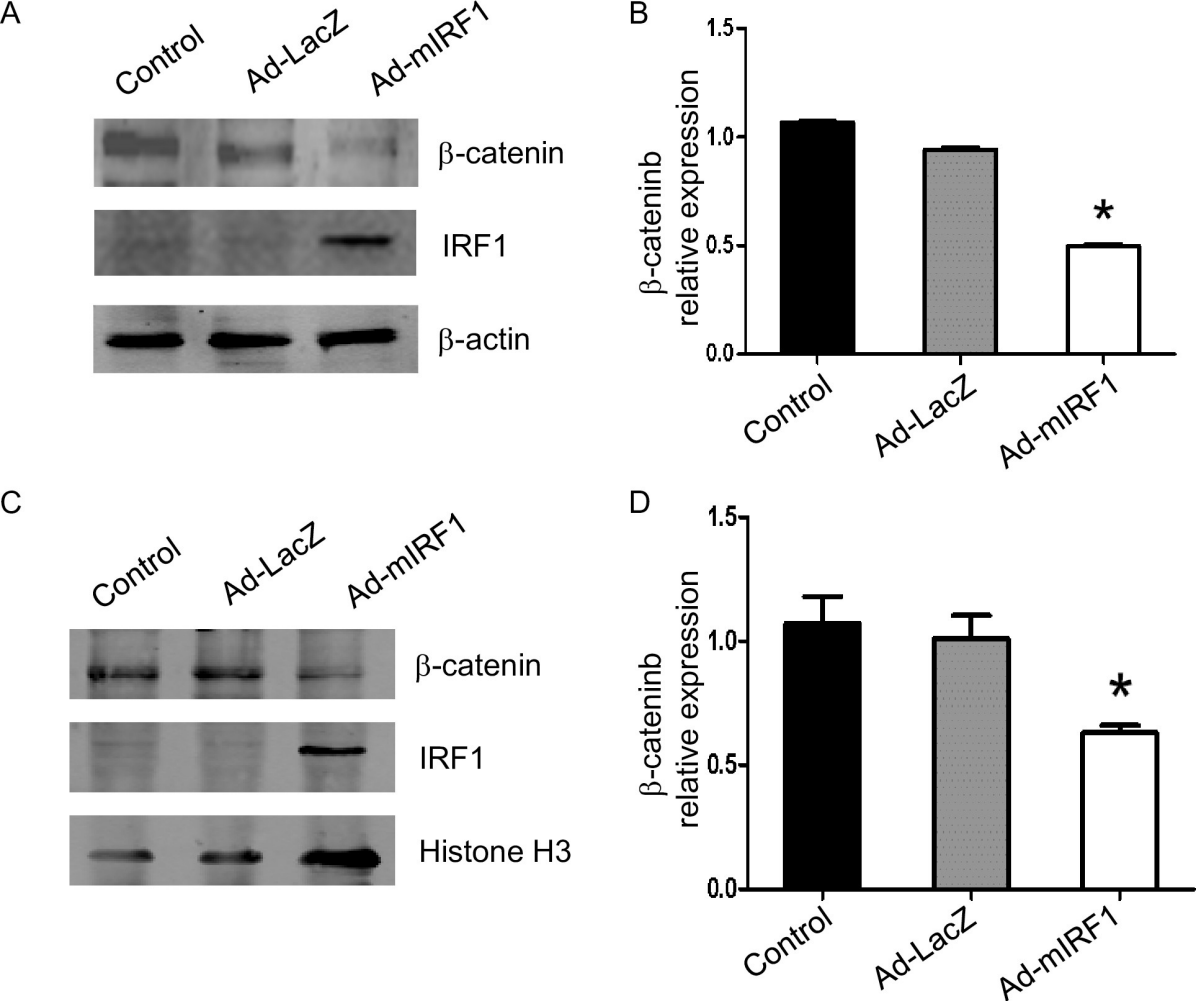
IRF1 inhibits β-catenin expression in primary liver cells. Primary liver cells of B6 mouse were infected with Ad-mIRF1 for 48 h and the cytoplasm protein (A and B) and nuclear proteins (C and D) of β-catenin and LC3A/B were detected by Western blot and densitometric analysis. n = 3, data are presented as mean±SD, *P< 0.05.

To explore the effect of IRF1, we transduced mouse hepatocytes with AdIRF1. As expected, AdIRF1 transduction resulted in strong cytoplasmic (Fig 5A) and nuclear (Fig 5C) IRF1 protein expression in naïve mouse hepatocytes. IRF1 expression significantly suppressed both cytoplasmic (Fig 5A and 5B) and nuclear (Fig 5C and 5D) β-catenin levels at 48 hrs. AdLacZ was used as control and did not modify β-catenin levels.

### IRF1 induces autophagy in mouse hepatocytes

**Fig 6:**
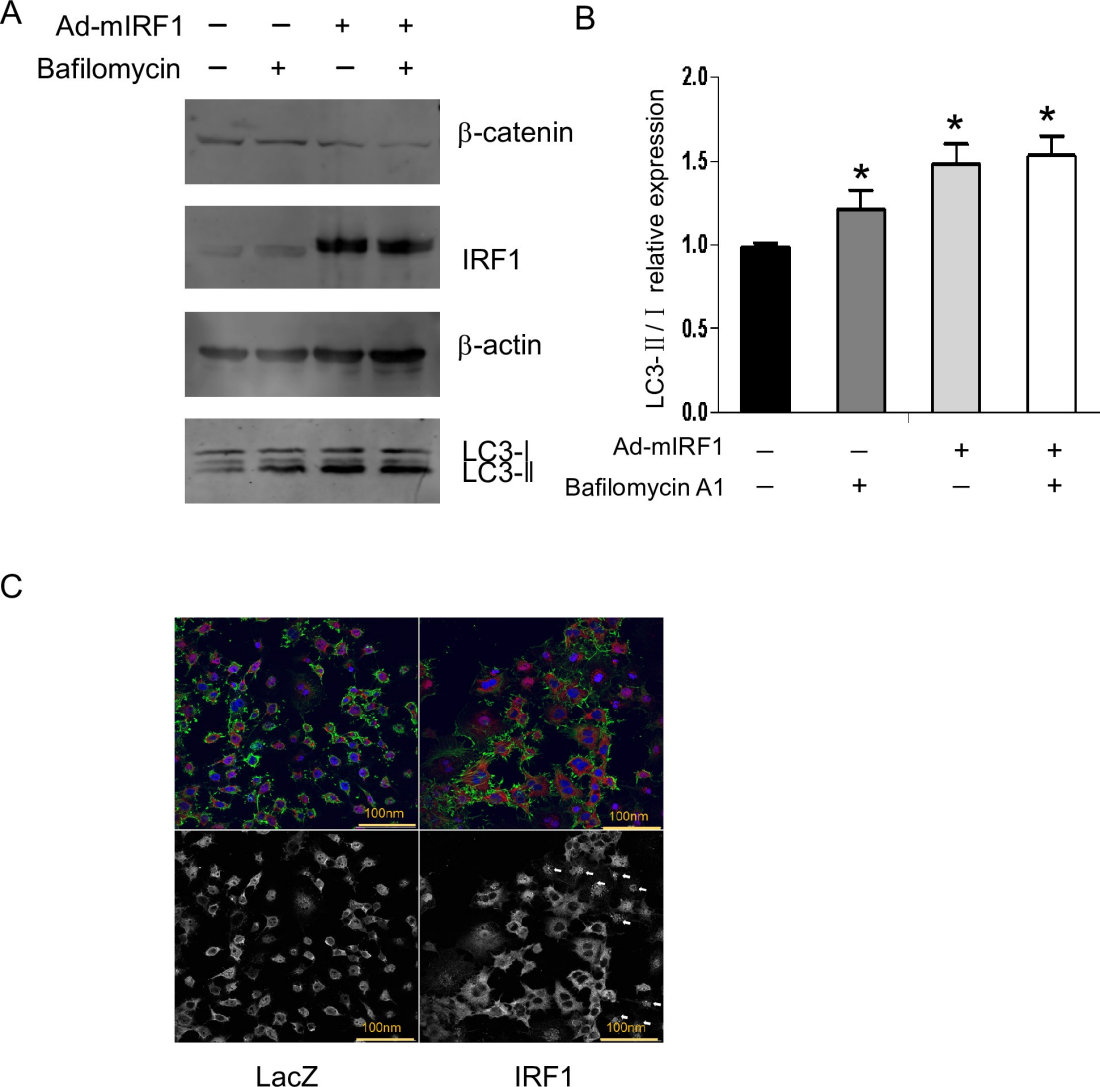
IRF1 induces autophagy in mouse primary liver cell. A and B) Primary B6 murine hepatocytes were infected with Ad-mIRF1 for 48 h and 40 nmol/L Bafilomycin A1 was added for an additional 4h. β-catenin, IRF1, and LC3 proteins were detected by Western Blot and densitometric analysis. C) Hepatocytes were infected with AdIRF1 or AdLacZ (as control) and then assayed by Immunofluorescence staining for Autophagy (Red is anti-LC3, Green is F-actin, Blue is nuclei. The lower pictures are the de-colored red light signal of the upper pictures, and the arrows are pointing to the puncta characteristic of autophagy) (Bars, 100 nm).

To examine the role of hepatocyte IRF1 in autophagy, we transduced mouse hepatocytes with AdIRF1. This resulted in high levels of IRF1 protein and was associated with decreased β-catenin protein levels on Western blot (Fig 6A). We then performed an LC3 turnover assay and found endogenous LC3-II was increased by IRF1 expression in primary hepatocytes (Fig 6A and 6B). Bafilomycin is an inhibitor of the late phase of autophagy and increased hepatocyte LC3-II expression. Addition of IRF1 to Bafilomycin further enhanced LC3-II protein levels, which shows that IRF1 enhances autophagy flux. To confirm that IRF1 induced autophagosome formation in hepatocytes, we transduced murine hepatocytes with AdIRF1 or AdLacZ as control. IRF1 expression increased autophagy puncta shown by immunofluorescence staining (Fig 6C, arrows in lower panel point to the puncta characteristic of autophagy).

### β-catenin inhibits autophagy

**Fig 7:**
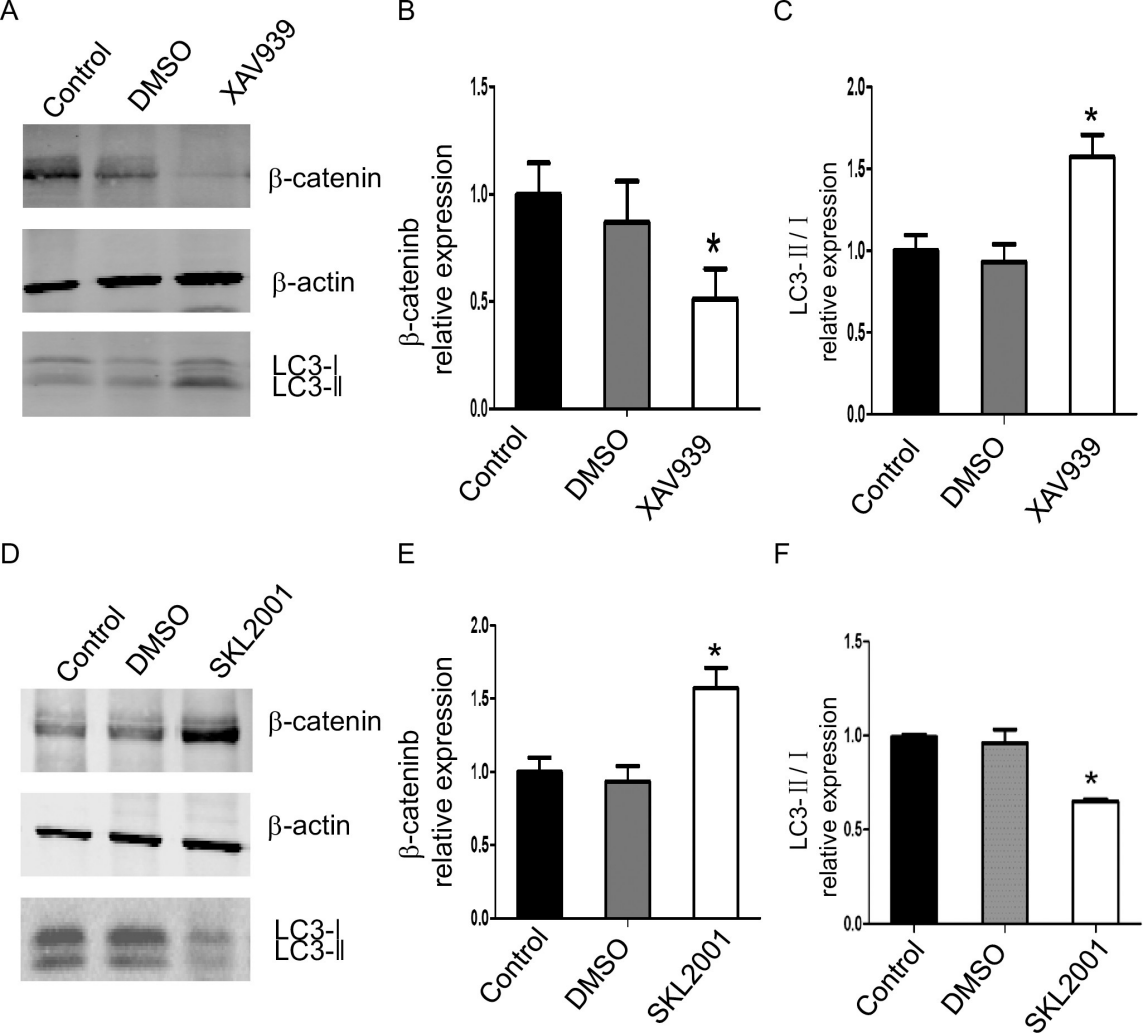
β-catenin inhibits autophagy in primary mouse hepatocytes. A, B, and C) Primary liver cells were treated with XAV939 or (D, E, and F) SKL2001 for 24 h and β-catenin and LC3 were detected by Western Blot and densitometric analysis. n = 3, data are presented as mean±SD, *P< 0.05.

To determine the role of β-catenin in autophagy, we used the β-catenin inhibitor XAV939 and β-catenin agonist SKL2001 in hepatocytes. In primary liver cells, XAV939 inhibited β-catenin protein levels, and this was associated with increased autophagy with elevated LC3-II levels compared to control (Fig 7A, 7B and 7C). The β-catenin agonist SKL2001 increased β-catenin protein levels, and this was associated with decreased autophagy with lower LC3-II levels compared to control (Fig 7D, 7E and 7F). These findings support an inhibitory effect of β-catenin on autophagy.

## Discussion

**Fig 8:**
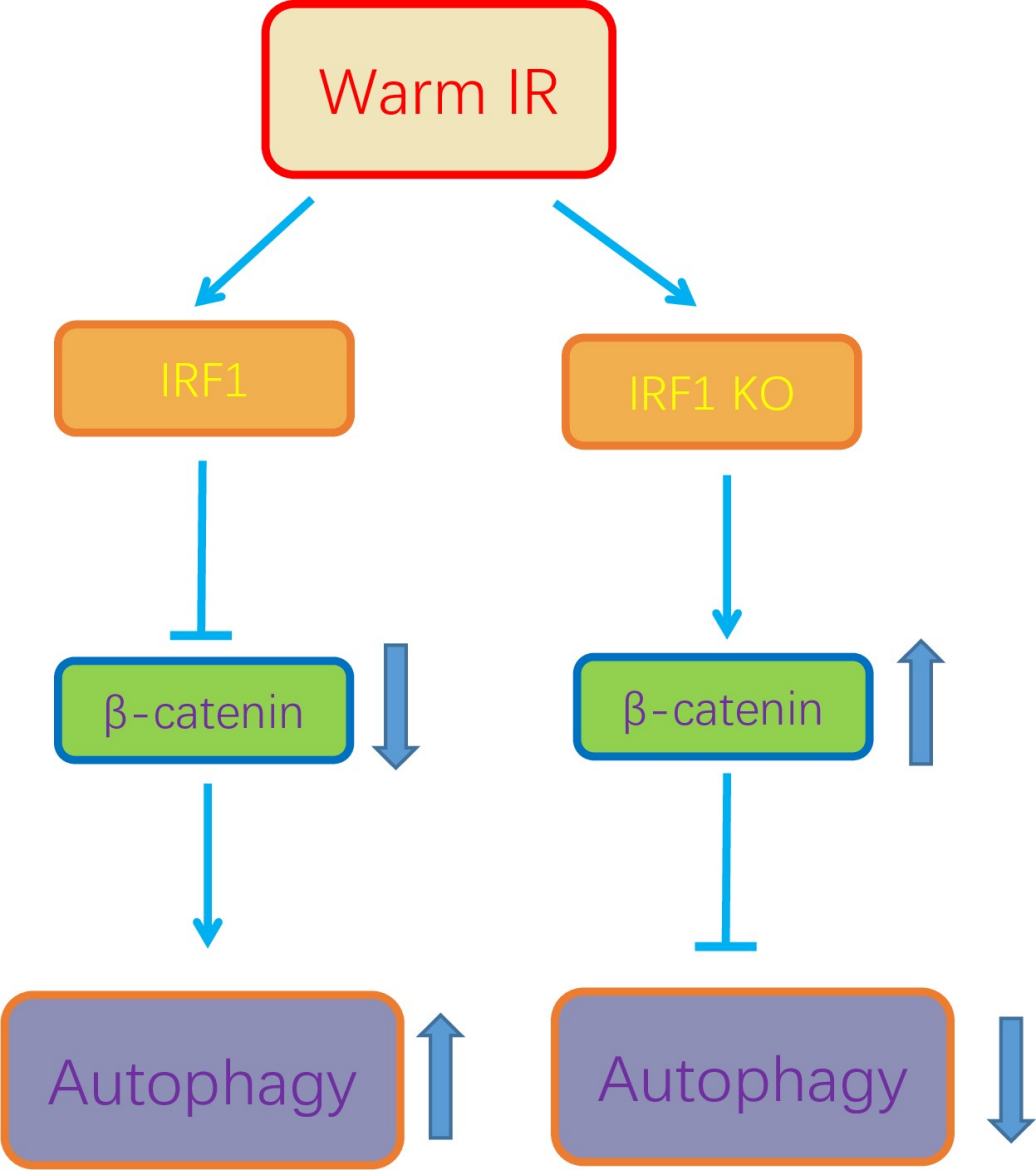
IRF1 activates autophagy to promote liver IR injury by inhibiting β-catenin. IRF1 is induced in hepatic IR and inhibits β-catenin and is associated with increased autophagy. In IRF1 KO mice, β-catenin expression is increased and is associated with decreased autophagy and less liver damage.

The major findings of this study are: (i) IRF1 was induced in hepatic IR injury; (ii) IRF1 activity induced autophagy during liver warm IR; (iii) IRF1 bound β-catenin protein and IRF1 expression decreased β-catenin in vitro and in vivo with IRF1 KO mice exhibiting increased hepatic β-catenin levels; (iv) Expression of IRF1 induced autophagy by inhibiting β-catenin. (v) β-catenin expression is inversely correlated with autophagy. Taken together, these findings demonstrate that IRF1 is induced in hepatic IR and inhibits β-catenin and is associated with increased autophagy. In IRF1 KO mice, β-catenin expression is increased and is associated with decreased autophagy and less liver damage (Fig 8).

Transcription factors within the IRF family play an important role in a variety of cellular responses including hematopoietic development, immune response and oncogenesis [21]. IRF1, is expressed at low levels in a plethora of cell types, including both parenchymal cells and nonparenchymal cells in the liver. IRF upregulation induced by Type I and II Interferons, viral infection, LPS in addition to several cytokines including TNF-a, IL-1β, IL6 [22] and IL23 [23]. IRF-1 has several targets including iNOS, IL-12p40 [24], and caspase 1. In a prior study we were able to show, that IRF-1 plays a key role in causing apoptosis of hepatocytes following cold I/R injury in syngeneic mice [12, 13, 25]. However, the role of hepatic IRF-1 in autophagy is not well understood. A study by Schwartz et al. has shown that IRF1 regulates the switch between autophagy and apoptosis in breast cancer cells [26]. However, during warm IR, IRF1 activates autophagy to aggravate hepatic ischemia-reperfusion injury via the P38/P62 pathway [16]. In our current study, we also found IRF1 induced autophagy in liver warm IR and autophagy showed harm to hepatocytes.

β-catenin also plays a key role in liver ischemia reperfusion injury [27, 28] and shows prospected to liver [29]. The β-catenin signaling pathway is important within hepatic development, regeneration and carcinogenesis [30]. The canonical Wnt signaling pathway is regulated through post-translational modifications of β-catenin [31]. Hepatocyte-specific β-catenin knockdown mice have been shown to be more sensitive to warm liver IR injury resulting in higher amount of necrosis and apoptosis [27]. We show that in mice hepatocyte β-catenin inhibited autophagy and may protect the hepatocytes in warm IR. Furthermore, we found that the IRF1 bound β-catenin and decreased β-catenin levels in hepatocytes. IRF1 gene and protein expression were markedly increased at 6 and 12 hr after IR and this matched reasonably well the timing of downstream suppressive effect on β-catenin protein in WT mice since the IRF1 KO liver showed increased β-catenin at 6 and 12 hrs. At 24 hr after IR, IRF1 gene expression is returning towards baseline, but the IRF1 protein level remained elevated. Meanwhile the inhibitory effect of IRF1 on β-catenin is gone by 24 hrs, which suggests this is a transient effect and other factors may also be regulating β-catenin expression or degradation. These results are consistent with the notion that IRF-1 decreases β-catenin during warm IR which increases autophagy, while the opposite occurs in the IRF1 KO mice.

In summary, this study provides novel evidence that IRF1 induces autophagy in part by inhibiting the β-catenin pathway during liver IR. Our observations have significant clinical relevance as liver IR is present during the majority of surgical procedures involving the liver. Future studies are needed to evaluate the role of IRF1 manipulation or other modulators of IRF1 signaling in clinically relevant settings involving a hepatic oxidative stress response.

## Supporting information

S1 Fig Liver damage is attenuated during warm ischemia-reperfusion (I/R) injury by treatment with 3-MA. (A and B) hematoxylin-eosin (HE) staining of liver tissue following warm I/R in 6h with treated 3-MA (15mg/kg i.p.) (A) or PBS (control) (B). (C and D) The serum ALT and AST levels were significantly increased in 3-MA treated mice after exposure to IR 6 hrs as compared with the control mice (n = 3). data are presented as mean±SD, *P< 0.05.
(PDF)

S1 Raw images (PDF)
