# Supplementary figures and images for: Interferon Regulatory Factor-1 (IRF1) activates autophagy to promote liver ischemia/reperfusion injury by inhibiting β-catenin in mice
Source: PLoS One. 2020 Nov 2;15(11):e0239119. doi: 10.1371/journal.pone.0239119 (PMC7605671; doi:10.1371/journal.pone.0239119)

S1 Fig

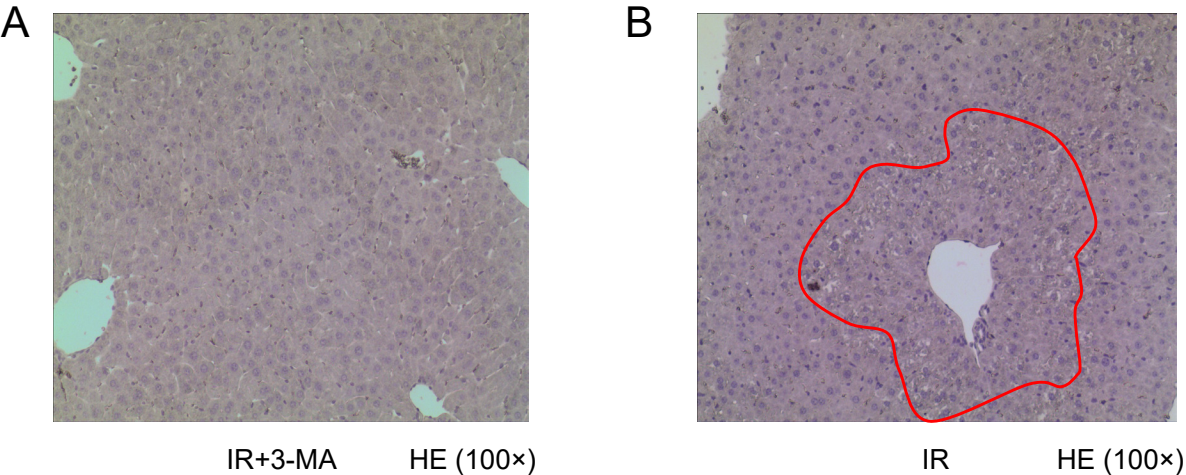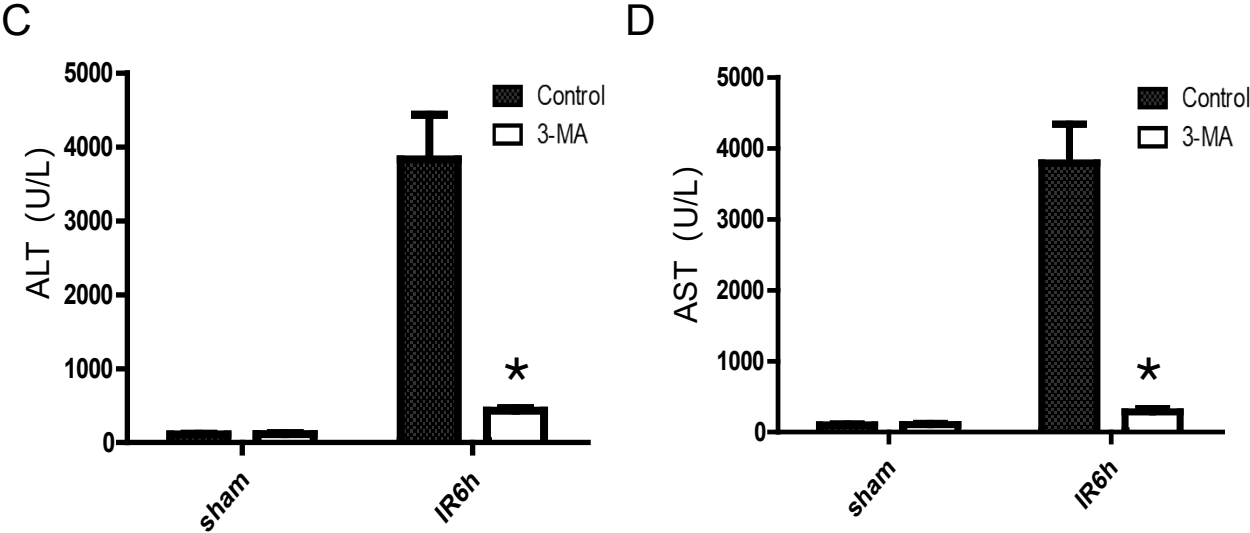

Supplement: S1 Fig — (A and B) hematoxylin-eosin (HE) staining of liver tissue following warm I/R in 6h with treated 3-MA (15mg/kg i.p.) (A) or PBS (control) (B). (C and D) The serum ALT and AST levels were significantly increased in 3-MA treated mice after exposure to IR 6 hrs as compared with the control mice (n = 3). data are presented as mean±SD, *P< 0.05. (PDF) [file pone.0239119.s001.pdf]

Figure 1

D

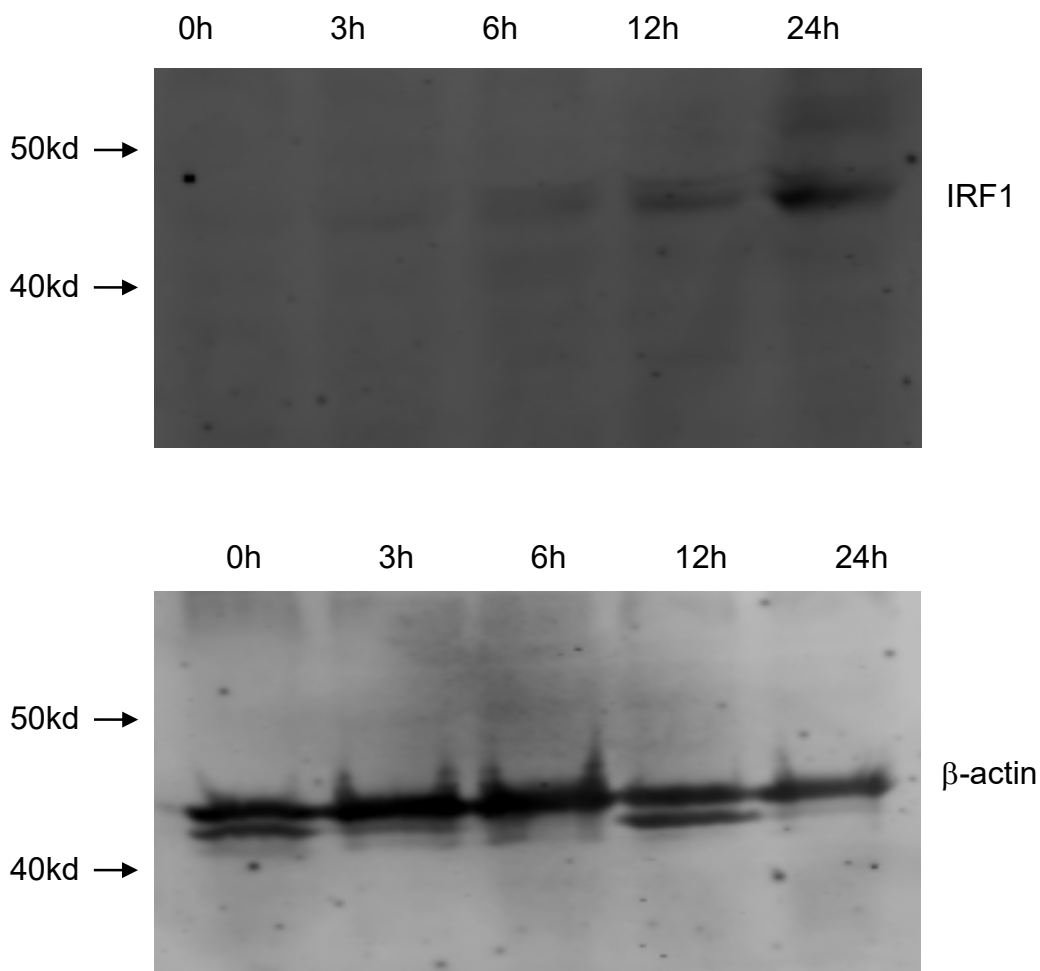

Figure 1

F

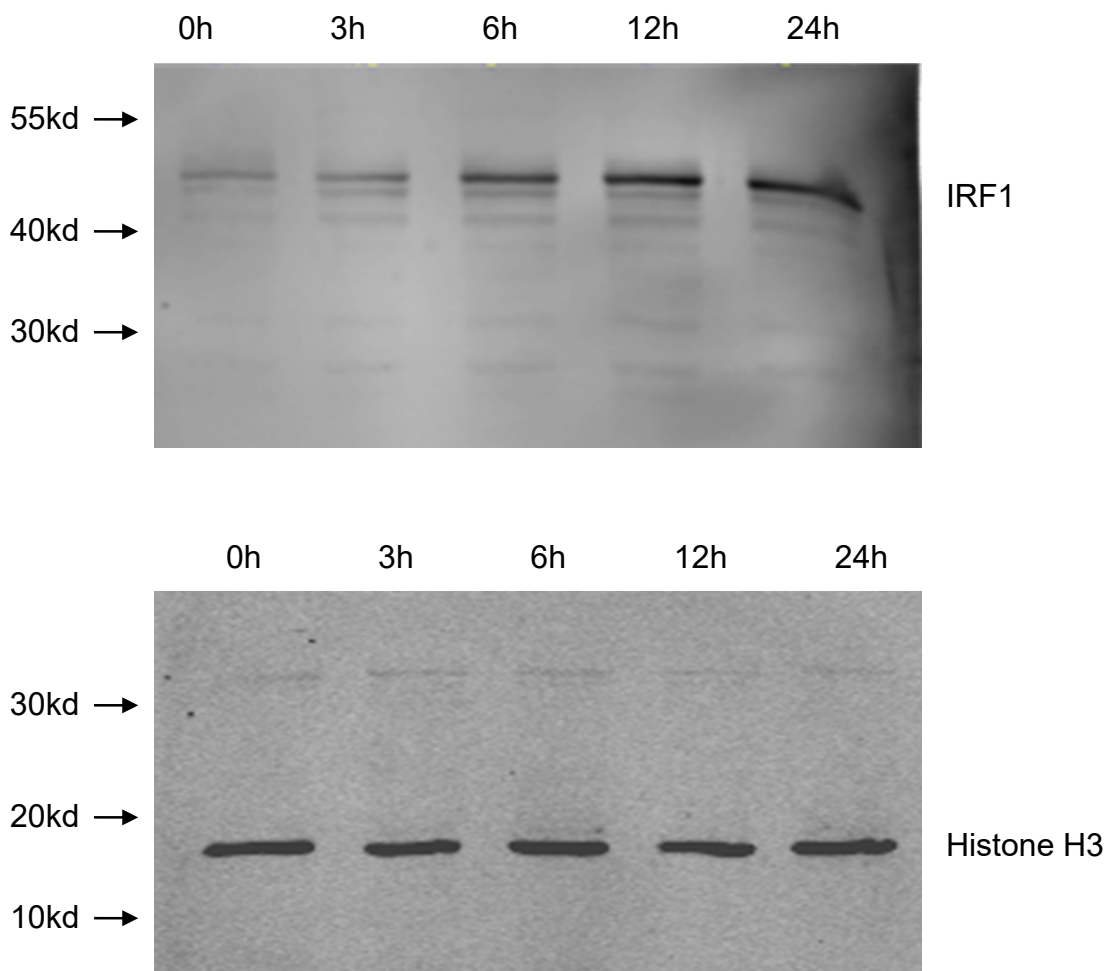

Figure 2

A

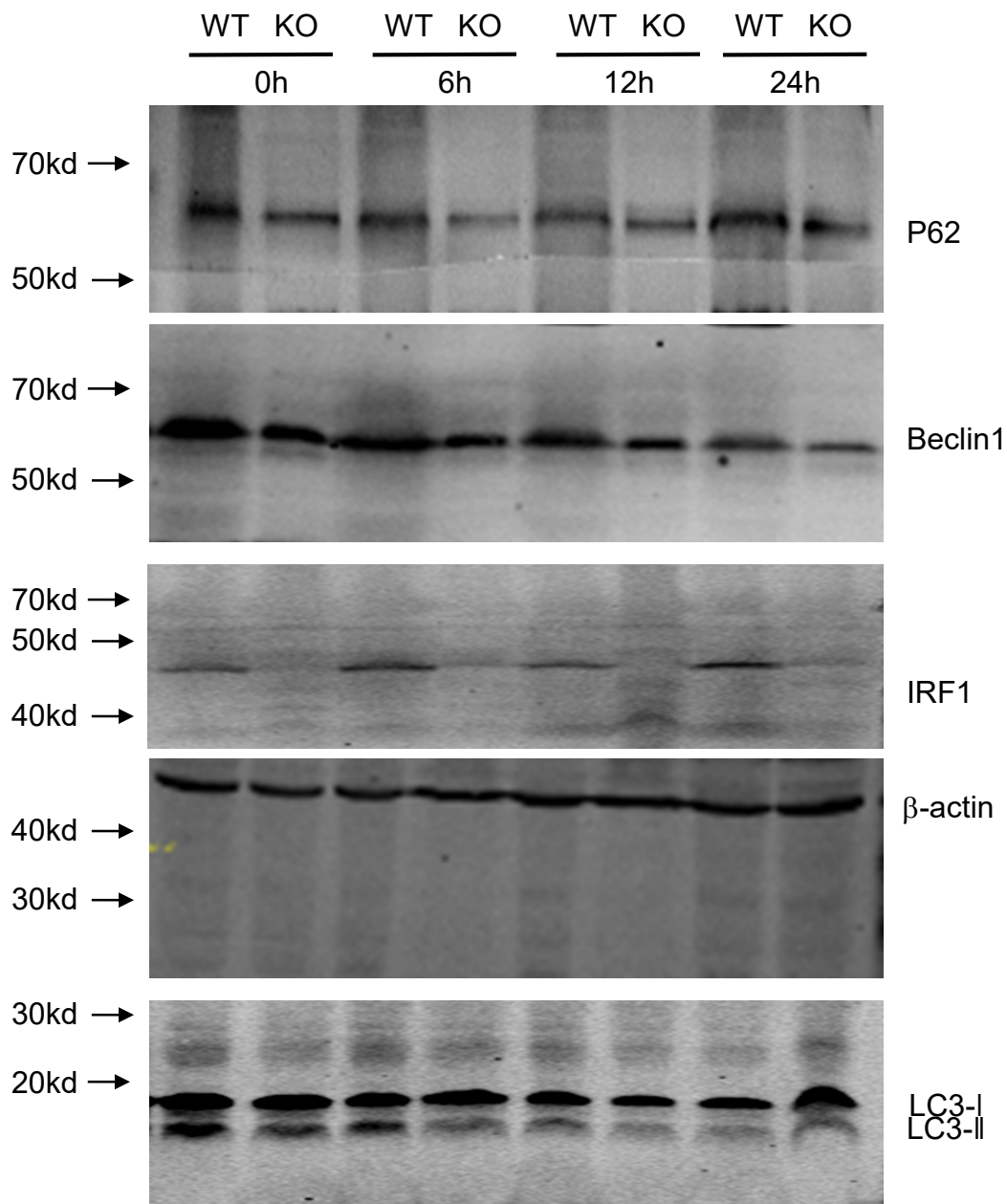

Figure 3

B

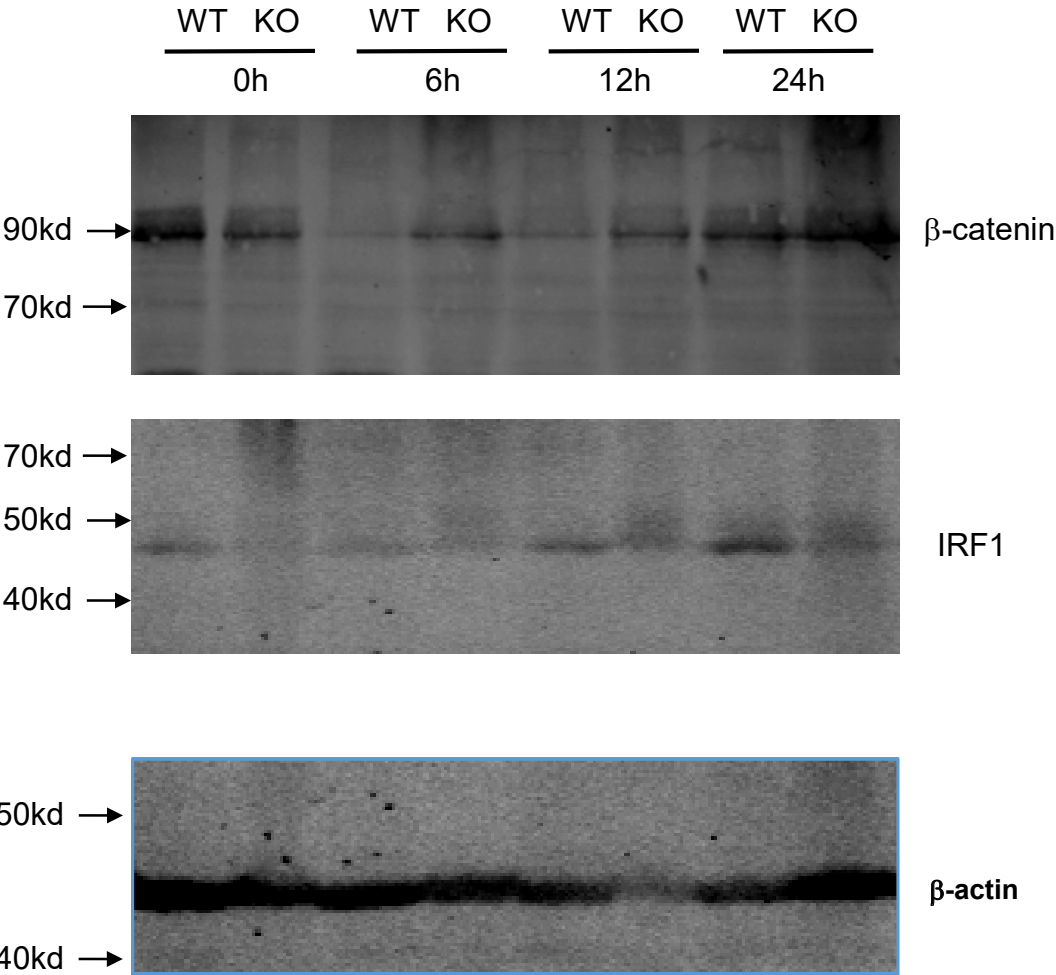

Figure 3

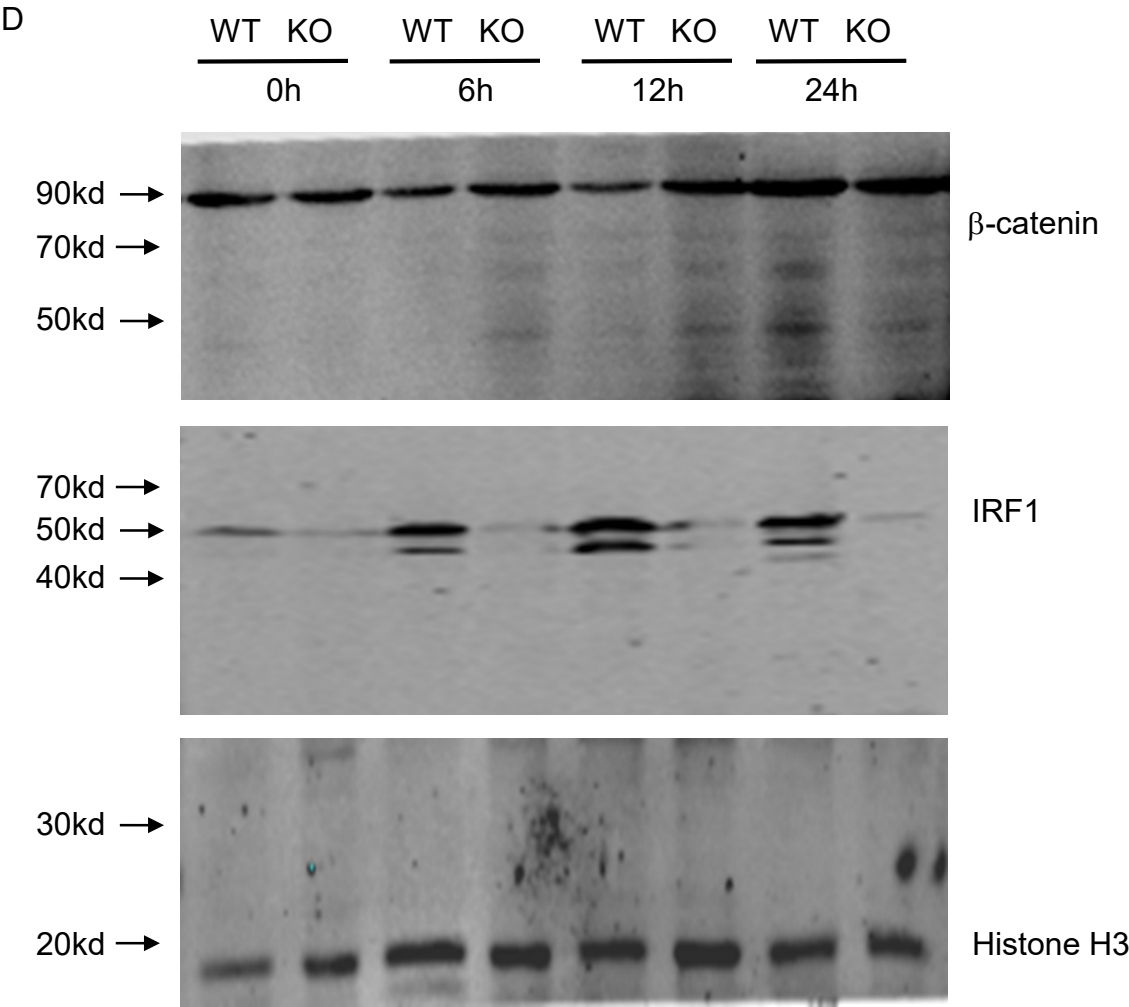

Figure 4

A

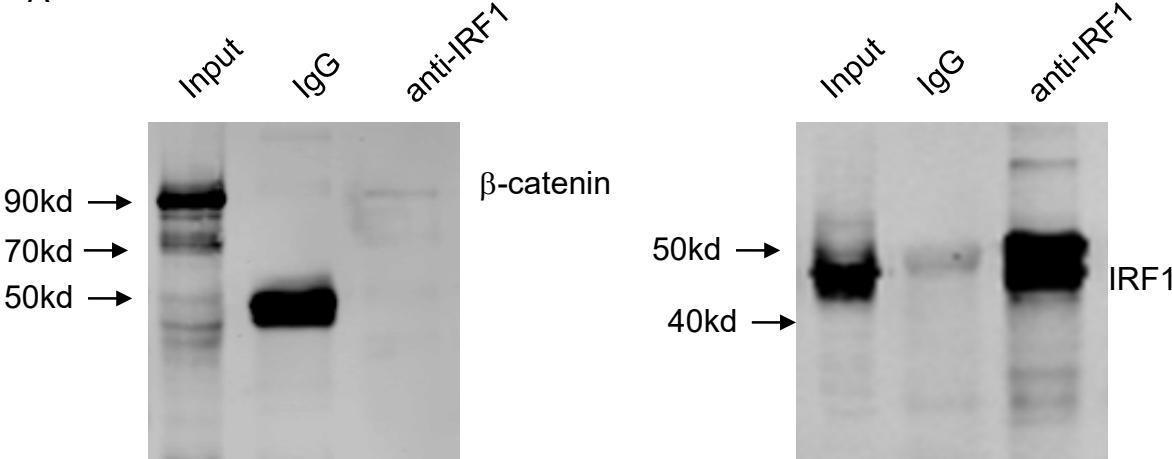

B

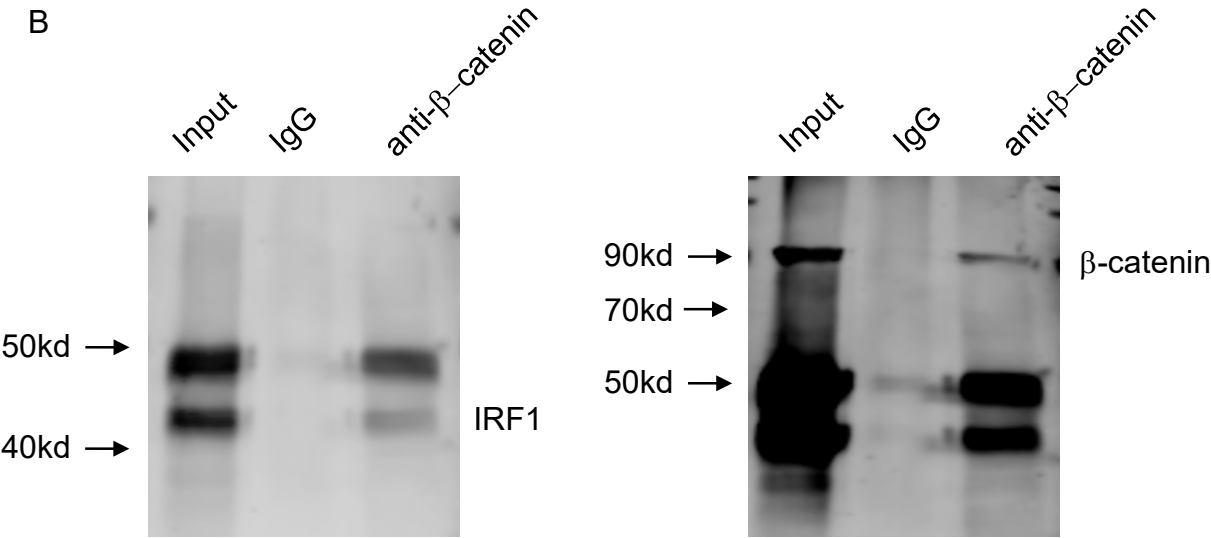

Figure 4

C

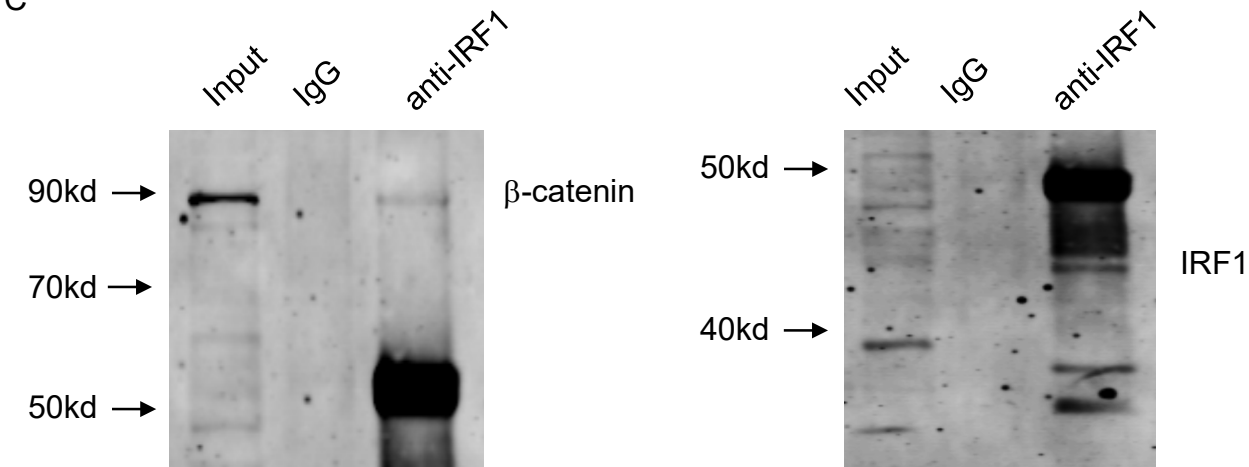

Figure 5

A

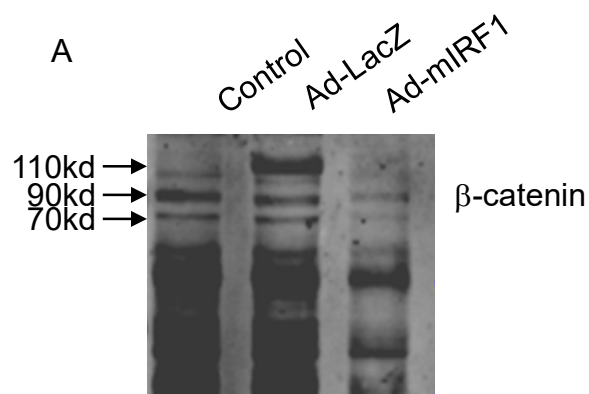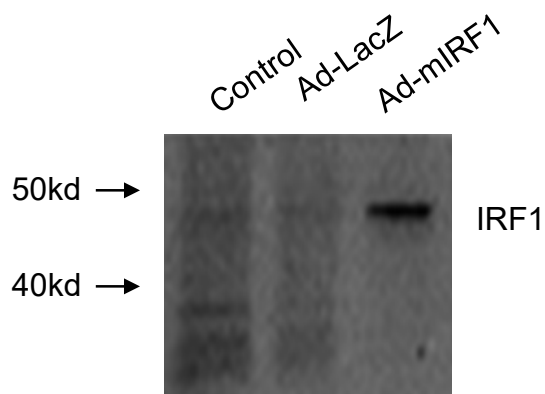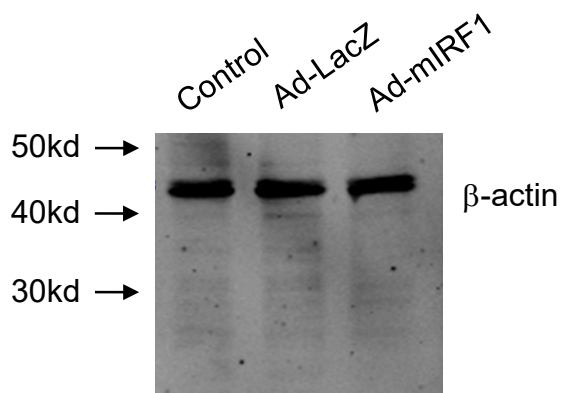

C

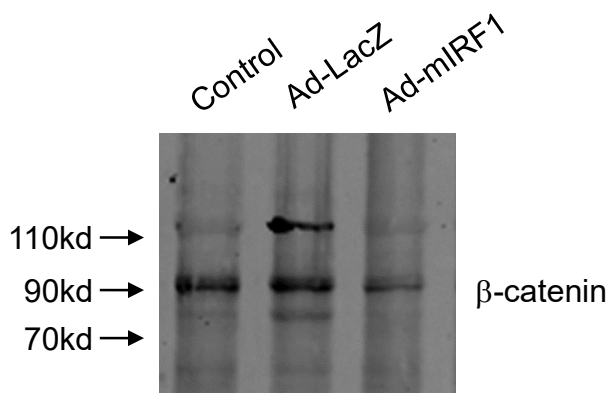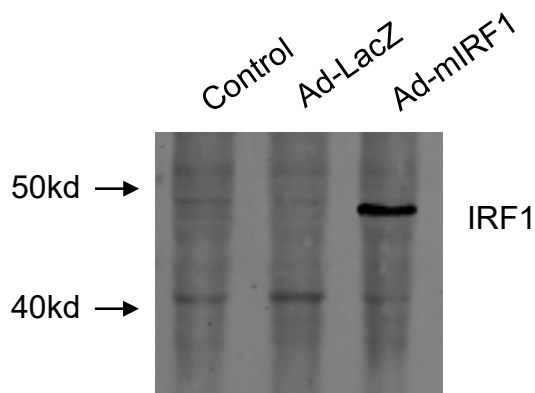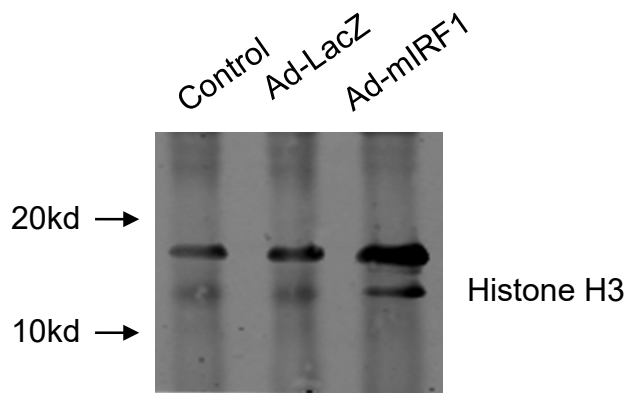

Figure 6

A

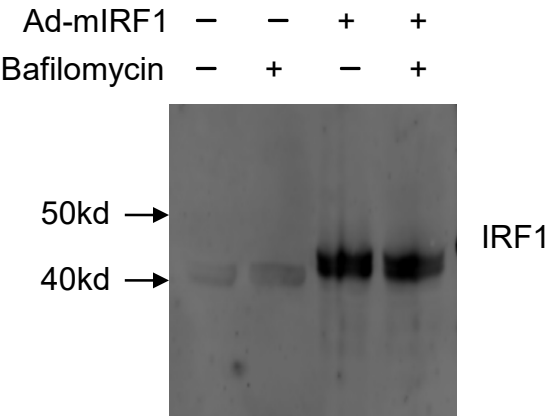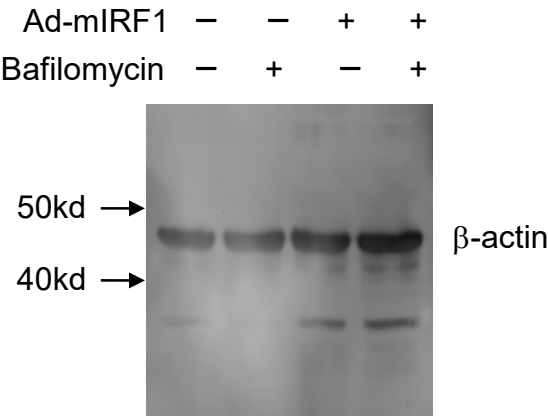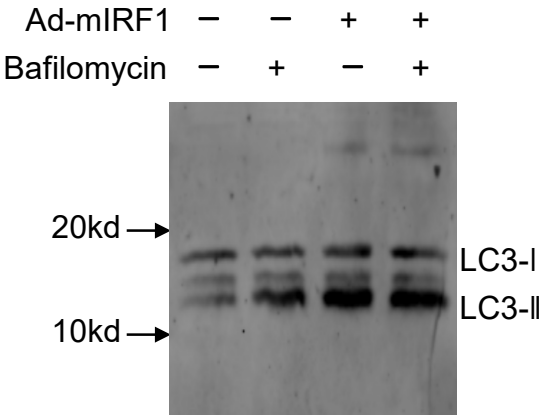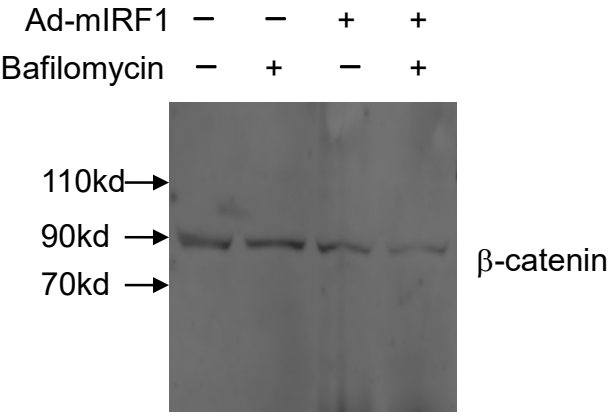

Figure 7

A

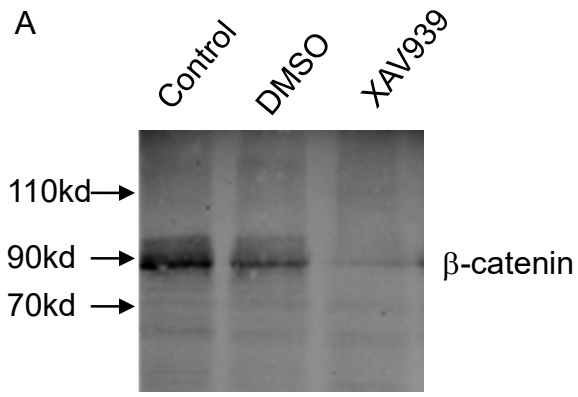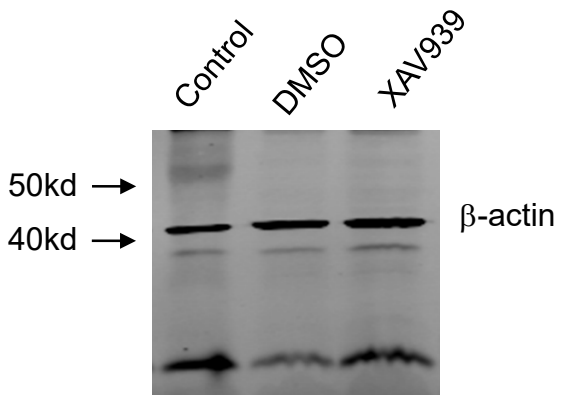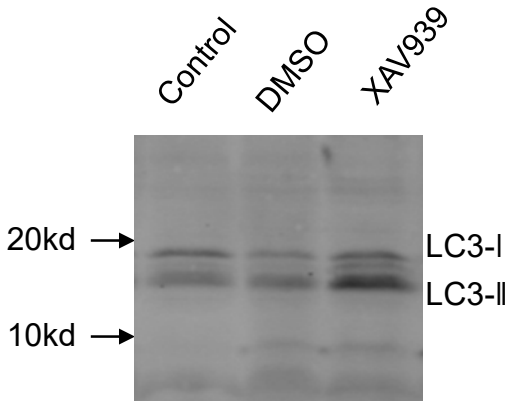

C

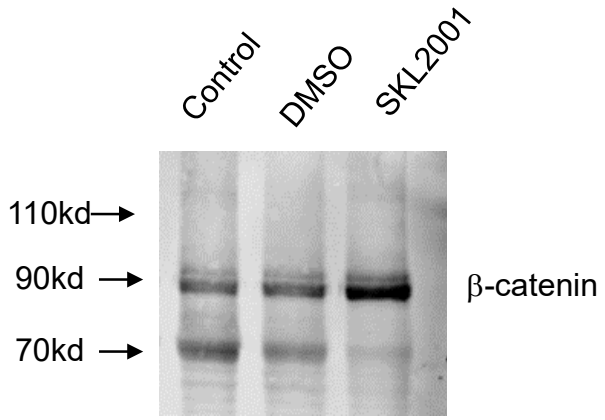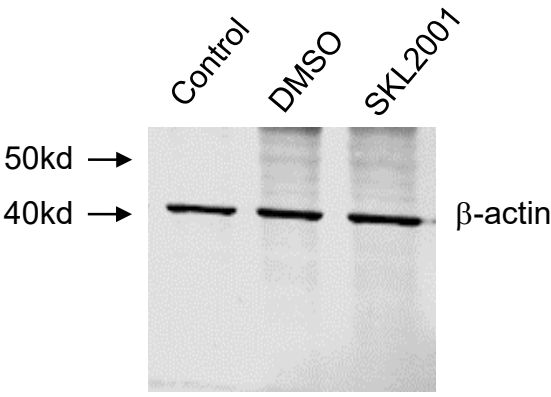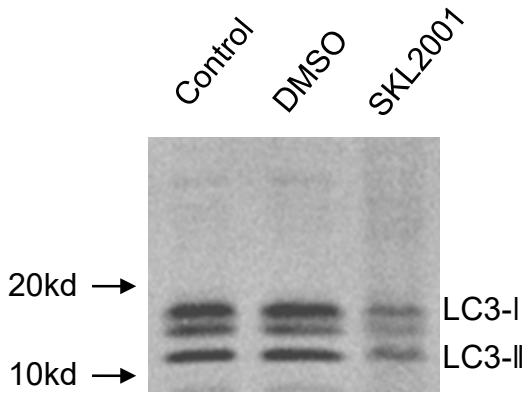

Supplement: S1 Raw images — (PDF) [file pone.0239119.s002.pdf]
